# PMiSLocMF: predicting miRNA subcellular localizations by incorporating multi-source features of miRNAs

**DOI:** 10.1093/bib/bbae386

**Published:** 2024-08-18

**Authors:** Lei Chen, Jiahui Gu, Bo Zhou

**Affiliations:** College of Information Engineering, Shanghai Maritime University, 1550 Haigang Avenue, Pudong New District, Shanghai 201306, China; College of Information Engineering, Shanghai Maritime University, 1550 Haigang Avenue, Pudong New District, Shanghai 201306, China; School of Basic Medical Sciences, Shanghai University of Medicine and Health Sciences, 279 Zhouzhu Road, Pudong New District, Shanghai 201318, China

**Keywords:** subcellular localization, miRNA, graph attention auto-encoder, node2vec, miRNA–drug association, miRNA–disease association, miRNA–mRNA association

## Abstract

The microRNAs (miRNAs) play crucial roles in several biological processes. It is essential for a deeper insight into their functions and mechanisms by detecting their subcellular localizations. The traditional methods for determining miRNAs subcellular localizations are expensive. The computational methods are alternative ways to quickly predict miRNAs subcellular localizations. Although several computational methods have been proposed in this regard, the incomplete representations of miRNAs in these methods left the room for improvement. In this study, a novel computational method for predicting miRNA subcellular localizations, named PMiSLocMF, was developed. As lots of miRNAs have multiple subcellular localizations, this method was a multi-label classifier. Several properties of miRNA, such as miRNA sequences, miRNA functional similarity, miRNA-disease, miRNA-drug, and miRNA–mRNA associations were adopted for generating informative miRNA features. To this end, powerful algorithms [node2vec and graph attention auto-encoder (GATE)] and one newly designed scheme were adopted to process above properties, producing five feature types. All features were poured into self-attention and fully connected layers to make predictions. The cross-validation results indicated the high performance of PMiSLocMF with accuracy higher than 0.83, average area under the receiver operating characteristic curve (AUC) and area under the precision-recall curve (AUPR) exceeding 0.90 and 0.77, respectively. Such performance was better than all previous methods based on the same dataset. Further tests proved that using all feature types can improve the performance of PMiSLocMF, and GATE and self-attention layer can help enhance the performance. Finally, we deeply analyzed the influence of miRNA associations with diseases, drugs, and mRNAs on PMiSLocMF. The dataset and codes are available at https://github.com/Gu20201017/PMiSLocMF.

## Introduction

MicroRNAs (miRNAs) are a class of short non-coding RNA molecules [1, 2], typically composed of ~20 nucleotides. They have been extensively studied due to their pivotal roles in cellular regulation [3–6]. The miRNAs are involved in the regulation of various biological processes, such as cell proliferation, differentiation and apoptosis, by binding to the mRNA of target genes [1, 7–9]. More and more studies show that the abnormal expression of miRNA is closely related to a variety of diseases, including cancers [10, 11], neurological diseases [12–14], and cardiovascular diseases [15–17]. The miRNAs influence the functional state of cells by regulating the expression of target genes at the post-transcriptional level and thus participate in the pathogenesis of diseases. Furthermore, recent studies reveal the close relationship between miRNAs and drugs [18, 19]. The miRNAs can be regulated by drugs and further served as potential drug targets. The mechanisms of miRNA function are complex and multifaceted. Temporary ‘pausing’ of mRNA translation by miRNA-induced silencing complex (RISC) is a major function of miRNAs, which occurs in the cytoplasm and processing bodies [20]. In addition, miRNAs have shown potential novel functions in other subcellular compartments. For example, miRNAs that mature in the cytoplasm can be translocated back to the nucleus. The most important function of these nuclear miRNAs is their ability to bind to complementary sequences on Cis-Regulatory Elements (CREs) (e.g., promoters [21–24] and enhancers [25–27]), thereby regulating transcriptional activation or repression of target genes [28]. miRNAs are also present in the nucleolus, where they are able to influence ribosomal RNA (rRNA) in the cell, which in turn exert their biological activities [29]. When miRNAs enter the mitochondria, they bind to mRNAs encoded by mtDNAs, enabling the regulation of mitochondria-related functions, especially in cancer cells [30]. Membrane-derived extracellular vesicles (EVs), such as microvesicles, exosomes, and extracellular vesicle, serve as key mediators of intercellular communication. Certain disease states have also identified a potential role for dysregulated EV-miRNA levels in pathogenesis. These diseases include chronic lung disease, immune response, neuroinflammation, diabetes, cancer and heart disease [31]. In view of the above, miRNAs have specific physiological roles at different cellular sites, and their subcellular localization is essential to gain insight into their physiological functions. Correct identification of miRNA subcellular localizations is helpful to advance the progresses in above research fields. Traditional experimental-based methods, such as classical *in situ* hybridization and high-throughput RNA sequencing techniques [32–34], to detect miRNA subcellular localizations are quite expensive. On the other hand, the huge number of detected miRNAs make them impossible to complete detection in time. Thus, it is urgent to develop quick and reliable methods to predict miRNA subcellular localizations.

In recent years, it is popular to design computational methods for investigating various biological problems. The newly proposed computer algorithms provide strong technique support for designing such methods. However, these methods are generally based on abundant data. Fortunately, more and more properties of miRNAs are uncovered, making it possible to use these properties to predict subcellular localizations. To date, some computational methods have been proposed to identify miRNA subcellular localizations. Most methods only used the miRNA sequence information [35–39]. Xiao et al. adopted a bidirectional long short-term memory (BiLSTM) to encode miRNA sequences and LSTM to decode the localization sets [35]. Asim et al. proposed the MirLocPredictor to identify miRNA subcellular localizations, which integrated a novel feature extraction scheme, named kmerPR2vec, to yield features from miRNA sequences [36]. Later, they built another method, named L2S-MirLoc, for the prediction of miRNA subcellular localizations [37]. The method adopted electronion interaction pseudoPotentials (EIIP) to obtain statistical representations of miRNA sequences and used data transformation approach to transform the multi-label miRNA subcellular localization problem into multi-class problems. Meher et al. designed the method, miRNALoc, for predicting miRNA subcellular localizations [38]. Features based on pseudo dinucleotide compositions (PseDNC) and di-nucleotide properties (DiPro) were extracted from miRNA sequences in this method, which were further transformed into principal component scores. Liang et al. designed the model MGFmiRNAloc for the prediction of miRNA subcellular localizations [39]. The model proposed simplified molecular input line entry system (SMILES) format to represent miRNA sequences and adopted graphical convolutional network (GCN) to access RNA sequence molecular map features. Some other methods were developed using different miRNA properties, such as gene ontology (GO) of miRNAs [40] and miRNA–mRNA associations [41]. Yang et al. first developed a novel scheme to measure the functional similarity for miRNAs based on their GO annotations and used the similarity score to predict miRNA subcellular localizations [40]. Xu et al. employed the associated mRNAs of miRNAs and their subcellular localizations to build the network method MiRLoc for detecting miRNA subcellular localizations [41]. Recently, Bai et al. proposed the method DAmiRLocGNet, which extracted miRNA features from sequences and miRNA–disease associations [42]. Its performance was better than MiRLoc in detecting miRNA subcellular localizations. Although the aforementioned methods shown good performance in determining miRNA subcellular localizations. An evident limitation exists. Most methods only adopted single miRNA properties to set up the method, inducing the incomplete representations of miRNAs. This incomplete representation influences the performance of the following prediction. The DAmiRLocGNet adopted sequences and miRNA-disease associations to represent miRNAs. However, it is still not enough to fully encode miRNAs. As mentioned above, miRNAs are highly associated with mRNAs [1, 7–9], diseases [10–17], and drugs [18, 19]. Employment of all this information is helpful to fully represent miRNAs, thereby setting up more powerful computational methods.

In this study, a novel multi-label classifier, named PMiSLocMF (predicting miRNA subcellular localizations with multi-source features), was developed for the prediction of miRNA subcellular localizations. Several properties of miRNAs, such as miRNA sequences, miRNA functional similarity, miRNA-disease, miRNA-drug and miRNA–mRNA (together with subcellular localizations of mRNAs) associations, were fused in the classifier. The powerful algorithms [node2vec [43] and graph attention auto-encoder (GATE) [44]] and a newly designed scheme processed above miRNA properties to yield five informative miRNA feature types. All these features were fed into self-attention and fully connected layers to make predictions. The cross-validation results shown that PMiSLocMF yielded accuracy higher than 0.83, average area under the receiver operating characteristic curve (AUC) and area under the precision-recall curve (AUPR) on seven subcellular localizations higher than 0.90 and 0.77, respectively. Such performance was better than all previous methods based on the same dataset. The classifier also had strong generalization ability by testing it on an independent test dataset. Further tests elaborated that all feature types provided positive contributions for predicting miRNA subcellular localizations, and GATE and self-attention layer can help improve the performance. Moreover, the influence of miRNA associations with diseases, drugs, and mRNAs on PMiSLocMF was deeply analyzed.

## Materials and methods

### Benchmark dataset

A well-defined dataset is very important for building efficient classifiers. In this study, we obtained the human miRNA subcellular localization dataset *S* from two previous studies [41, 42], which was originally retrieved from RNALocate database (version 2.0) [45]. There are 1041 miRNAs in this dataset. These miRNAs are all in the functional similarity network constructed in [41] and are classified into seven subcellular localizations, including cytoplasm, exosome, nucleolus, nucleus, extracellular vesicle, microvesicle, and mitochondrion. Each localization contained more than 50 miRNAs. For formulation, the miRNAs having one subcellular location constitute a subset, denoted by $S\left(\text{cytoplasm}\right)$, $S\left(\text{exosome}\right)$, $S\left(\text{nucleolus}\right)$, $S\left(\text{nucleus}\right)$, $S\left(\text{extracellular}\ \text{vesicle}\right)$, $S\left(\text{microvesicle}\right)$, $S\left(\text{mitochondrion}\right)$  *.* Then, the benchmark dataset *S* can be formulated by.


(1) \begin{align*} &S=S\left(\text{cytoplasm}\right)\cup\ S\left(\text{exosome}\right)\cup\ S\left(\text{nucleolus}\right)\nonumber\\ &\qquad\cup S\left(\text{nucleus}\right)\cup\ S\left(\text{extracellular}\ \text{vesicle}\right)\cup\ S\left(\text{microvesicle}\right)\nonumber\\ &\qquad\cup S\left(\text{mitochondrion}\right) \end{align*}

**Figure 1:**
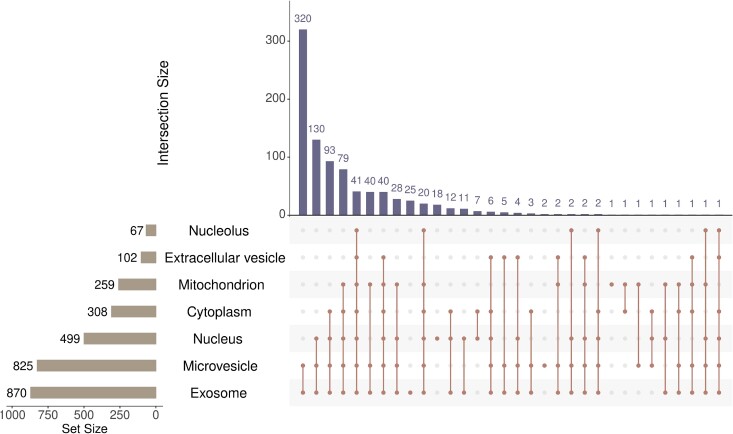
Upset graph to show the intersections of human miRNAs in seven subcellular localizations. Lots of miRNAs have multiple subcellular localizations.

As some miRNAs can have multiple subcellular localizations, the intersection of some above subsets contains common miRNAs. To illustrate this fact, an upset graph was plotted, as shown in Fig. 1. It can be observed that miRNAs having exosome are the most (870) and those having nucleolus are the least (67). Lots of miRNAs have multiple subcellular localizations, where 41 miRNAs have all seven localizations. Thus, when subcellular localizations are deemed as labels and miRNAs are regarded as samples, it is a multi-label classification problem to classify miRNAs into subcellular localizations.

In addition, we constructed an independent test dataset from RNALocate database (version 2.0) [45] to test the generalization ability of the proposed model. All human miRNAs with subcellular localizations in RNALocate database were downloaded. After removing above 1041 miRNAs, 2793 miRNAs were obtained. Then, we further removed the miRNAs without disease association information, accessing 41 miRNAs. Their distribution on seven subcellular localizations is listed in Table 1. It can be observed that the sum of miRNA numbers in seven localizations was large than 41, implying some miRNAs can belong to more than one subcellular localization, which was same as those in dataset *S*.

**Table 1:** Distribution of 41 miRNAs in the independent test dataset on seven subcellular localizations.

Subcellular location	Number of miRNAs
Cytoplasm	8
Exosome	36
Nucleolus	1
Nucleus	11
Extracellular vesicle	1
Microvesicle	36
Mitochondrion	8

### Multi-source properties of miRNAs

Recently, it is very popular to employ multiple properties of objects for building efficient classifiers. To date, several miRNA properties have been uncovered and collected in some public databases, making it possible for predicting miRNA subcellular localizations using these properties. This study adopted multiple properties of miRNAs, which are introduced as below.

#### miRNA-disease association network

In recent years, lots of evidences have been reported that miRNAs have strong associations with diseases. Diseases can be deemed as the functions of miRNAs and have been used to predict miRNA subcellular localizations [42]. This study adopted the miRNA-disease associations collected in [42], involving 1041 miRNAs, 640 diseases, and 15 547 miRNA-disease associations. In fact, these associations were extracted from the miRNA-disease associations reported in human miRNA disease database (HMDD, version 3.2) [46]. Based on this association information, an association network was built. The 1041 miRNAs and 640 diseases were termed as nodes. One miRNA-disease association determined one edge in this network. For convenience, this network was denoted by ${N}_{mi- di}$.

In addition, above miRNA-disease associations can be represented by a matrix *MD*, where rows represented 1041 miRNAs and columns indicated 640 diseases. $MD\left({m}_i,{d}_j\right)=1$ if miRNA ${m}_i$ and disease ${d}_j$ can constitute a miRNA-disease association; otherwise, it was set to zero. Based on this matrix, the Gaussian interaction profile (GIP) kernel similarity between two miRNAs ${m}_i$ and ${m}_j$ was calculated by


(2) \begin{equation*} GM\left({m}_i,{m}_j\right)=\exp \big(-{\lambda}_m\parallel MD\big({m}_i\big)- MD\big({m}_j\big){\parallel}^2\big)\big), \end{equation*}

where ${\lambda}_m={n}_m/{\sum}_{k=1}^{n_m}{\left\Vert MD\left({m}_k\right)\right\Vert}^2$ (${n}_m$ was the number of miRNAs), $MD\left({m}_i\right)/ MD\left({m}_j\right)$ was the row of *MD* representing ${m}_i/{m}_j$.

#### miRNA sequences and similarity network

The sequences of miRNAs are always the first-hand information for investigating miRNAs. They were also used in this study. For all miRNAs mentioned in Section Benchmark dataset, their sequences were sourced from miRBase database [47]. The Smith–Waterman algorithm [48] was adopted to measure the similarity of two miRNA sequences. For miRNAs ${m}_i$ and ${m}_j$, their sequence similarity can be computed by


(3) \begin{equation*} SW\left({m}_i,{m}_j\right)=\frac{sp\left({m}_i,{m}_j\right)}{\sqrt{sp\left({m}_i,{m}_i\right)\cdot sp\left({m}_j,{m}_j\right)}}, \end{equation*}

where $sp\left({m}_i,{m}_j\right)$ stands for the local alignment score of two sequences. For convenience, the similarity score between any two miRNA sequences was downloaded from the previous study [42]. As lots of outcomes of Eq. 3 were zero, we used miRNA GIP kernel similarity for supplement, formulated as


(4) \begin{equation*} SW\_ GM\left({m}_i,{m}_j\right)=\left\{\begin{array}{@{}cc} SW\left({m}_i,{m}_j\right)&\ \text{if}\ SW\left({m}_i,{m}_j\right)>0\ \\{} GM\left({m}_i,{m}_j\right)&\ \text{others}\ \end{array}\right. \end{equation*}

Based on the outcomes of Eq. 4, a miRNA sequence similarity network was constructed, denoted by ${N}_S$. The 1041 miRNAs were defined as nodes, whereas two miRNAs were connected if and only if their sequence similarity score was larger than zero. Furthermore, the similarity score was assigned to the corresponding edge as its weight.

#### miRNA-drug association network

Like diseases, drugs have also been confirmed to be related to miRNAs. The drugs related to one miRNA may be helpful to predict its subcellular localizations. This information was adopted in this study to construct the classifier. The miRNA-drug associations were retrieved from ncDR [49], a comprehensive chemoinformatics and bioinformatics resource collecting curated and predicted non-coding RNAs associated with drug resistance. After restricting to 1041 miRNAs, 3305 miRNA-drug associations were obtained. These associations involved 130 drugs. Accordingly, another association network containing 1041 miRNAs and 130 drugs as nodes was set up. The miRNA-drug associations determined the edges in this network. Let us denote this network as ${N}_{mi- dr}$.

#### miRNA functional similarity network

In addition to sequence similarity network, we also employed the miRNA functional similarity network. The Wang et al.’s method was adopted to access the functional similarity of miRNAs [50], which was based on the diseases of miRNAs. In medical subject heading (MeSH), all diseases are placed in an acyclic graph (DAG). The edges in this DAG indicates the children or parents of diseases. Each disease *di* can be represented by a subgraph containing it and all its ancestor nodes. The contribution of a disease *dt* in this subgraph to the disease *di* is defined as


(5) \begin{equation*} \left\{\begin{array}{@{}l}{D}_{di}(dt)=1\kern18.15em if\ dt= di\\{D}_{di}(dt)=\max \left\{\Delta \cdot{D}_{di}\left(d{t}^{\prime}\right):d{t}^{\prime }\ is\ a\ child\ of\ dt\right\}\kern1.75em if\ dt\ne di\ \end{array}\right., \end{equation*}

where $\Delta$ is a parameter, which was suggested to set 0.5 [50]. Then, the semantic value of disease *di* is computed by


(6) \begin{equation*} DS(di)=\sum_{dt\in A(di)}{D}_{di}(dt), \end{equation*}

where $A(di)$ is a set containing *di* and all its ancestor nodes. Then, the semantic similarity of two diseases $di$ and ${di}^{\prime }$ is defined as


(7) \begin{equation*} SS\left( di,{di}^{\prime}\right)=\frac{\sum_{dt\in A(di)\cap A\left(di^{\prime}\right)}\left({D}_{di}(dt)+{D}_{d{i}^{\prime }}(dt)\right)}{DS(di)+ DS\left({di}^{\prime}\right)}, \end{equation*}

For two miRNAs ${m}_i$ and ${m}_j$, their functional similarity can be measured according to their related to diseases. The diseases related to ${m}_i$ constitute the disease set $DD\left({m}_i\right)$ and that for ${m}_j$ is denoted as $DD\left({m}_j\right)$. The relationship between $DD\left({m}_i\right)$ and $DD\left({m}_j\right)$ can indicate the functional similarity of ${m}_i$ and ${m}_j$, which can be calculated by. 


(8) \begin{equation*} mFS\left({m}_i,{m}_j\right)=\frac{\sum_{di\in DD\left({m}_i\right)}S\left( di, DD\left({m}_j\right)\right)+{\sum}_{di\in DD\left({m}_j\right)}S\left( di, DD\left({m}_i\right)\right)}{\left| DD\left({m}_i\right)\right|+\left| DD\left({m}_j\right)\right|}, \end{equation*}

where $S\left( di, DD\left({m}_j\right)\right)=\max \left\{ SS\left( di,d{i}^{\prime}\right):{di}^{\prime}\in DD\left({m}_j\right))\right\}$. Likewise, above similarity was fused with miRNA GIP kernel similarity (Eq. 2) to reduce the phenomenon that lots of outcomes of Eq. 8 were zero, defined by


(9) \begin{equation*} mFS\_ GM\left({m}_i,{m}_j\right)=\left\{\begin{array}{@{}cc} mFS\left({m}_i,{m}_j\right)&\ \text{if}\ mFS\left({m}_i,{m}_j\right)>0\ \\{} GM\left({m}_i,{m}_j\right)&\ \text{others}\ \end{array}\right. \end{equation*}

The miRNA functional similarity network was constructed with the above definition on functional similarity of any two miRNAs. It defined 1041 miRNAs as nodes and the edges in it were determined by a binarization threshold *T*. If the outcome of Eq. 9 was larger than *T*, the corresponding miRNAs were adjacent. The obtained miRNA functional similarity network was denoted by ${N}_F$.

#### miRNA–mRNA association network and subcellular localizations of mRNAs

mRNAs are a widely studied type of RNAs. Their subcellular localizations have been well-studied. On the other hand, the special associations between miRNAs and mRNAs have been partly revealed in recent years. One previous study confirmed that the subcellular localization data of miRNA target mRNAs are helpful to identify the subcellular localizations of miRNAs [41]. Thus, we also adopted the information on miRNA–mRNA associations and subcellular localizations of mRNAs to construct the classifier.

The miRNA–mRNA associations were obtained from Xu et al.’s study [41]. This information contained 8254 associations, involving 1041 miRNAs and 2836 mRNAs, which was extracted from the database miRTarBase 2020 [51] and screened by using immunohistochemistry, luciferase reporter assay, northern blot, qRT-PCR or another experimental method. After that, we built the third association network, which defined 1041 miRNAs and 2836 mRNAs as nodes and 8254 associations as edges. This network was denoted by ${N}_{mi-m}$.

**Figure 2:**
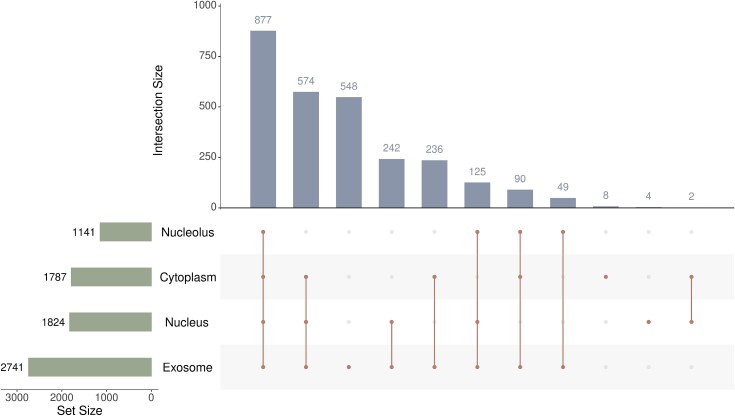
Upset graph to show the intersections of human mRNAs in four subcellular localizations. Many mRNAs have multiple subcellular localizations.

Besides the miRNA–mRNA association network, we also employed the subcellular localizations of above 2836 mRNAs, which were also used in Xu et al.’s study [41]. However, only four subcellular localizations: cytoplasm, exosome, nucleolus, and nucleus, contained more than 50 mRNAs. Other three subcellular localizations (extracellular vesicle, microvesicle, and mitochondrion) contained less than 50 mRNAs, which were removed for data validity. Likewise, some mRNAs have multiple subcellular localizations. Figure 2 shows the intersections of four mRNA subsets, corresponding to four subcellular localizations. mRNAs having exosome are the most (2741) and 877 mRNAs have all four localizations.

In this section, several properties of miRNAs were employed for constructing the classifier, including three association networks (Table 2), miRNA sequence and functional similarity networks. In addition, the subcellular localizations of mRNAs were also fused in the classifier to improve the performance.

**Table 2:** Overview of three association networks for miRNAs in dataset *S*.

Association network	Number of miRNAs	Number of other objects	Number of associations
miRNA-disease	1041	640 (diseases)	15,547
miRNA-drug	1041	130 (drugs)	3305
miRNA–mRNA	1041	2836 (mRNAs)	8254

### Multi-source features of miRNAs

Several properties of miRNAs were introduced in Section Multi-source properties of miRNAs. How to perfectly fuse them into the classifier is a challenging problem. Some existing or newly designed methods were adopted to tackle these properties. Informative miRNA features were obtained with the help of these methods.

#### Features yielded by node2vec

Network is now a popular form to investigate various problems, as the three miRNA association networks, miRNA sequence and functional similarity networks used in this study. To tackle such non-Euclidean data, some powerful methods have been proposed. Among them, network embedding algorithms are an important member, which can assign a numeric vector to each node in one or more networks. Node2vec is one of the most widely used network embedding algorithms [43]. Its detailed description is available in Supplementary Material I. In our study, we employed node2vec to extract miRNA features from miRNA sequence similarity network, miRNA-disease, miRNA-drug, and miRNA–mRNA association networks. In detail, from miRNA sequence similarity network ${N}_S$, a 64-D (dimension) vector was obtained for each miRNA through node2vec; whereas node2vec produced a 128-D vector for each miRNA from miRNA-disease, miRNA-drug, and miRNA–mRNA association networks, respectively.

#### Features yielded by GATE

In addition to network embedding algorithms, GCN [52] is another representative method to process networks. It can learn a new representation of each node from its raw representation and the given network. Here, a special GCN, GATE [44], was employed to yield high-level miRNA features. GATE contains encoder and decoder procedures. The encoder updates the raw representation of each node by considering the representations of its neighbors. The decoder tries to recover the raw representations of nodes as perfect as possible. The detailed description of GATE is provided in Supplementary Material I.

In present study, we applied GATE to the raw miRNA features derived from miRNA-disease, miRNA-drug, and miRNA–mRNA association networks through node2vec. The miRNA functional similarity network ${N}_F$ was also fed into GATE. Finally, we obtained three 128-D feature vectors of each miRNA, one is the improved features derived from miRNA-disease association network, the second one is obtained from miRNA-drug association network, and last one contains high-level features derived from miRNA–mRNA association network.

#### Features derived from miRNA–mRNA association network and subcellular localizations of mRNAs

In Xu et al.’s study [41], they have validated that interacting miRNAs and mRNAs tend to be localized to the same subcellular localizations. Furthermore, they demonstrated that incorporating the subcellular localization information of target mRNAs can help improve the performance of their model. Here, we designed a scheme to extract essential features from miRNA–mRNA associations and subcellular localizations of mRNAs.

Given a miRNA ${m}_i$, its target mRNAs can be extracted from the miRNA–mRNA association network, i.e., the neighbors of ${m}_i$ in this network. They constitute the mRNA set, denoted by $M\left({m}_i\right)$. As mentioned in Section miRNA–mRNA association network and subcellular localizations of mRNAs, four subcellular localizations are assigned to these mRNAs: cytoplasm, exosome, nucleolus, and nucleus. Accordingly, mRNAs in $M\left({m}_i\right)$ can be classified into four subsets, denoted by ${M}_{\text{cytoplasm}}\left({m}_i\right)$, ${M}_{\text{exosome}}\left({m}_i\right)$, ${M}_{\text{nucleolus}}\left({m}_i\right)$, and ${M}_{\text{nucleus}}\left({m}_i\right)$, respectively, where ${M}_{\text{cytoplasm}}\left({m}_i\right)$ contains the mRNAs in $M\left({m}_i\right)$ that have subcellular localization cytoplasm, and other three subsets are defined in a similar way. If one subset is obviously larger than other three subsets, ${m}_i$ has the corresponding subcellular localization with a high probability. However, directly using the sizes of above four subsets is not a perfect manner. We refine them as follows:


(10) \begin{equation*} \left\{\begin{array}{@{}c}{R}_{\text{cytoplasm}}\left({m}_i\right)=\displaystyle\frac{\left|{M}_{\text{cytoplasm}}\left({m}_i\right)\right|}{\left|M\left({m}_i\right)\right|}\\{}{R}_{\text{exosome}}\left({m}_i\right)=\displaystyle\frac{\left|{M}_{\text{exosome}}\left({m}_i\right)\right|}{\left|M\left({m}_i\right)\right|}\kern1.25em \\{}{R}_{\text{nucleolus}}\left({m}_i\right)=\displaystyle\frac{\left|{M}_{\text{nucleolus}}\left({m}_i\right)\right|}{\left|M\left({m}_i\right)\right|}\kern0.5em \\{}{R}_{\text{nucleus}}\left({m}_i\right)=\displaystyle\frac{\left|{M}_{\text{nucleus}}\left({m}_i\right)\right|}{\left|M\left({m}_i\right)\right|}\kern2em \end{array}\right.\kern-8pt, \end{equation*}

Under this operation, outcomes of Eq. 10 are all between 0 and 1, which is more proper to represent miRNA ${m}_i$. Thus, four features are obtained from miRNA–mRNA association network and subcellular localizations of mRNAs for each miRNA.

With above procedures, each miRNA can be encoded by five feature types. The first feature type was obtained from miRNA sequence similarity network through node2vec. The second feature type was derived from miRNA-disease association network through node2vec and was further processed by GATE and miRNA functional association network. The third and fourth feature types were similar to the second one, which were derived from miRNA-drug and miRNA–mRNA association networks, respectively, rather than miRNA-disease association network. The last feature type was derived from miRNA–mRNA association network. To obtain more informative features, the information of mRNA subcellular localizations was also employed to generate this feature type. For convenience, above five feature types were called miRNA sequence, disease, drug, mRNA network and mRNA co-localization features. The details of these five feature types are listed in Table 3.

**Table 3:** Breakdown of miRNA features used in PMiSLocMF.

Feature type	miRNA properties	Methods	Dimension
miRNA sequence features	miRNA sequence similarity network	Node2vec	64
miRNA disease features	miRNA-disease association network, miRNA functional similarity network	Node2vec, graph attention auto-encoder	128
miRNA drug features	miRNA-drug association network, miRNA functional similarity network	Node2vec, graph attention auto-encoder	128
miRNA mRNA network features	miRNA–mRNA association network, miRNA functional similarity network	Node2vec, graph attention auto-encoder	128
miRNA mRNA co-localization features	miRNA–mRNA association network, subcellular localizations of mRNAs	Newly designed scheme	4
Total			452

### Outline of the PMiSLocMF

**Figure 3:**
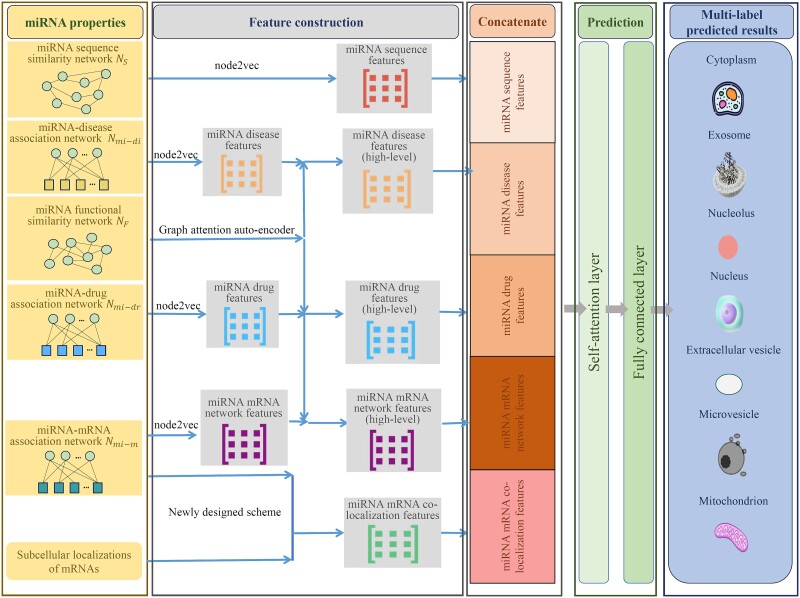
Entire procedures for constructing PMiSLocMF. Several miRNA properties are employed, which are used to extract miRNA features through node2vec, graph attention auto-encoder, and a newly designed scheme. All obtained miRNA features are concatenated and fed into the self-attention and fully connected layers to make predictions.

In this study, we utilized multiple miRNA properties derived from different sources to build the multi-label classifier, named PMiSLocMF, for the prediction of miRNA subcellular localizations. The entire procedures are illustrated in Fig. 3.

As mentioned in Section Multi-source properties of miRNAs, several miRNA properties, including miRNA sequence and functional similarity networks, miRNA-disease, miRNA-drug, and miRNA–mRNA association networks, were employed. With these properties, five feature types were generated to represent each miRNA. All these feature types were concatenated into one feature vector, which was fed into the prediction procedure. This procedure contained two parts: self-attention layer and fully connected layer. In the self-attention layer, self-attention mechanisms learn the weights between features for better representing the internal structure of the input features, which can help the model capture complex dependencies between features (see Supplementary Material I for detail). The feature vector processed by the self-attention layer were subjected to the fully connected layer, which contained two hidden layers and one output layer. Rectified linear unit (ReLU) activation functions were employed for two hidden layers and the activation function of output layer was sigmoid. The loss function was the binary cross-entropy, which is defined as.


(11) \begin{equation*} L=-\sum_i\kern0.1em \left[{y}_i\log \left({\hat{y}}_i\right)+\left(1-{y}_i\right)\log \left(1-{\hat{y}}_i\right)\right] \end{equation*}

where ${y}_i$ represents the observed label, ${\hat{y}}_i$ stands for the predicted probability. The trainable parameters in self-attention and fully connected layers were optimized by Adam optimizer [53]. The multi-label classifier was implemented by TensorFlow 2.11.0 and Scikit-learn [54].

### Evaluation metrics

Cross-validation is a commonly used method to assess the performance of classifiers [55]. Here, we adopted 10-fold cross-validation to evaluate the performance of classifiers. The cross-validation results were counted as several measurements, including aiming, coverage, accuracy, absolute true, and absolute false [56–65]. Besides the overall measurements, we also employed the popular receiver operating characteristic (ROC) and precision-recall (PR) curves for each label, along with the area under these two curves, denoted by AUC and AUPR. The detailed descriptions of above measurements are provided in Supplementary Material I.

## Result and discussion

### Performance of PMiSLocMF

The parameters of PMiSLocMF were tuned as described in Supplementary Material I and the optimal parameters are provided in Table S1. PMiSLocMF with optimized parameters was evaluated by 10-fold cross-validation. It was found that the aiming, coverage, accuracy, absolute true, and absolute false were 0.9067, 0.9087, 0.8374, 0.5000, and 0.1012, respectively. The aiming and coverage exceeded 0.9 and accuracy researched 0.8, indicating the good performance of PMiSLocMF.

**Figure 4:**
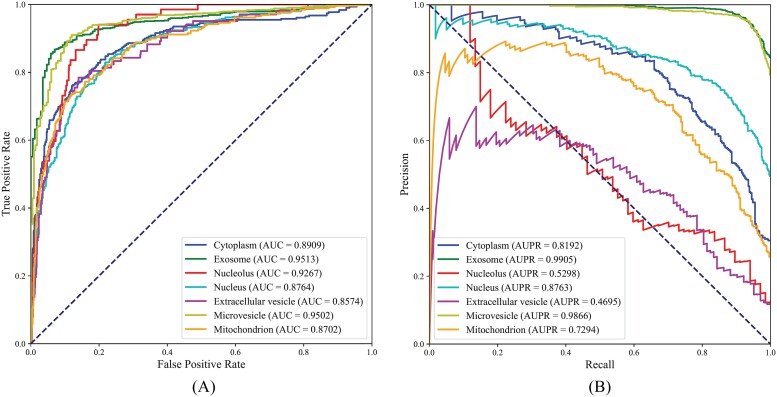
Performance of PMiSLocMF on seven subcellular localizations measured by ROC and PR curves. (A) ROC curves; (B) PR curves. All AUC values are high (>0.85) and four AUPR values are high (>0.8).

In addition, we also counted the performance of PMiSLocMF on seven subcellular localizations, measured by AUC and AUPR. The ROC and PR curves on seven localizations are shown in Fig. 4. The AUC values for cytoplasm, exosome, nucleolus, nucleus, extracellular vesicle, microvesicle, and mitochondrion were 0.8909, 0.9513, 0.9267, 0.8764, 0.8574, 0.9502, and 0.8702, respectively. These values were all higher than 0.85, suggesting the good performance of PMiSLocMF on each subcellular localization. As for AUPR values, they were 0.8192, 0.9905, 0.5298, 0.8763, 0.4695, 0.9866, and 0.7294. The AUPR values on cytoplasm, exosome, nucleus, microvesicle exceeded 0.8, whereas those on nucleolus and extracellular vesicle were not satisfied, slightly higher or lower than 0.5. By observing Fig. 1, the sizes of nucleolus and extracellular vesicle were smallest. When considering the performance of PMiSLocMF on them, the negative samples were much more than positive samples. AUPR is more sensitive to the imbalanced problem than AUC. All these induced the low AUPR values on these two subcellular localizations. The average AUC and AUPR were further calculated, yielding average AUC of 0.9033 and average AUPR of 0.7716.

### Effectiveness of five feature types

The proposed classifier PMiSLocMF used five feature types: miRNA sequence, disease, drug, mRNA network and mRNA co-localization features. Generally, the classifier can provide better performance if more related features are adopted. In this section, some ablation tests on features were conducted to prove the contribution of each feature type to PMiSLocMF and more features can yield better performance.

Five feature types can yield thirty combinations except that used in PMiSLocMF. For each feature type combination, a classifier was built and evaluated by 10-fold cross-validation. The overall performance of these thirty classifiers is listed in Table 4. The performance of PMiSLocMF is also listed in this table for easy comparisons. It was found that PMiSLocMF, which used all five feature types, provided the highest aiming, coverage, accuracy, absolute true, average AUC, and average AUPR, whereas it gave the lowest absolute false. These results indicated that using all feature types made the classifier more powerful. In addition, we also counted the performance of above thirty classifiers on seven subcellular localizations. The AUC and AUPR values are provided in Tables 5 and 6, respectively. The same measurements yielded by PMiSLocMF are also given in these two tables for easy comparisons. It can be observed that PMiSLocMF provided the highest AUC values on four localizations (exosome, nucleus, microvesicle, and mitochondrion) and also yielded the highest AUPR values on four localizations (exosome, nucleus, extracellular vesicle, and microvesicle). Such results suggested that PMiSLocMF was superior to thirty classifiers. As other thirty classifiers lacked one or more feature types, each feature type provided contributions for PMiSLocMF.

**Table 4:** Ablation test results on features (overall performance).

Ablation settings					Aiming	Coverage	Accuracy	Absolute true	Absolute false	Average AUC	Average AUPR
α	β	γ	δ	ε							
√					0.8809	0.8701	0.7712	0.3877	0.1371	0.8517	0.7193
	√				0.8759	0.8663	0.7579	0.3677	0.1449	0.7746	0.6729
		√			0.8724	0.8699	0.7546	0.3633	0.1466	0.7908	0.6647
			√		0.8588	0.8079	0.7046	0.3144	0.1801	0.7619	0.6202
				√	0.8558	0.8036	0.7052	0.3122	0.1788	0.7746	0.5859
√	√				0.8812	0.8707	0.7778	0.3911	0.1370	0.8685	0.7374
√		√			0.8847	0.8876	0.7766	0.3922	0.1276	0.8716	0.7454
√			√		0.8820	0.8723	0.7747	0.3977	0.1279	0.8775	0.7344
√				√	0.8808	0.8698	0.7723	0.3966	0.1341	0.8813	0.7428
	√	√			0.8834	0.8755	0.7733	0.3877	0.1355	0.8299	0.7203
	√		√		0.8784	0.8724	0.7574	0.3688	0.1449	0.8289	0.7139
	√			√	0.8758	0.8725	0.7608	0.3611	0.1425	0.8489	0.7205
		√	√		0.8780	0.8682	0.7627	0.3733	0.1426	0.8355	0.6904
		√		√	0.8762	0.8648	0.7528	0.3633	0.1461	0.8510	0.7048
			√	√	0.8595	0.8075	0.7070	0.3144	0.1739	0.7647	0.6091
√	√	√			0.8888	0.8789	0.7826	0.4044	0.1303	0.8837	0.7486
√	√		√		0.8828	0.8766	0.7746	0.3955	0.1306	0.8862	0.7464
√	√			√	0.8846	0.8714	0.7768	0.3944	0.1341	0.8901	0.7604
√		√	√		0.8854	0.8791	0.7829	0.4055	0.1273	0.8868	0.7612
√		√		√	0.8879	0.8746	0.7802	0.4100	0.1300	0.8974	0.7594
√			√	√	0.8827	0.8633	0.7675	0.3800	0.1355	0.8794	0.7499
	√	√	√		0.8840	0.8765	0.7766	0.3844	0.1311	0.8515	0.7283
	√	√		√	0.8864	0.8804	0.7816	0.4055	0.1277	0.8691	0.7502
	√		√	√	0.8782	0.8646	0.7574	0.3588	0.1453	0.8405	0.7247
		√	√	√	0.8876	0.8702	0.7733	0.3977	0.1339	0.8443	0.7102
√	√	√	√		0.8922	0.8919	0.8108	0.4788	0.1196	0.8977	0.7560
√	√	√		√	0.8894	0.8881	0.8007	0.4666	0.1226	0.9006	0.7662
√	√		√	√	0.8957	0.8796	0.7891	0.4111	0.1347	0.8882	0.7498
√		√	√	√	0.8887	0.8808	0.8085	0.4322	0.1261	0.8886	0.7598
	√	√	√	√	0.8903	0.8782	0.7894	0.4077	0.1314	0.8683	0.7423
√	√	√	√	√	**0.9067**	**0.9087**	**0.8374**	**0.5000**	**0.1012**	**0.9033**	**0.7716**

α: miRNA sequence features; β: miRNA disease features; γ: miRNA drug features; δ: miRNA mRNA network features; ε: miRNA mRNA co-localization features

**Table 5:** Ablation test results on features (performance on seven subcellular localizations measured by AUC).

Ablation settings					Cytoplasm	Exosome	Nucleolus	Nucleus	Extracellular vesicle	Microvesicle	Mitochondrion
α	β	γ	δ	ε							
√					0.8567	0.8491	0.9059	0.8363	0.8419	0.8365	0.8358
	√				0.8095	0.7055	0.8686	0.7676	0.7899	0.6836	0.7974
		√			0.8177	0.7790	0.8556	0.8007	0.7533	0.7657	0.7636
			√		0.7543	0.7819	0.7913	0.6990	0.7520	0.7801	0.7749
				√	0.7506	0.8089	0.7955	0.6995	0.7708	0.8109	0.7864
√	√				0.8551	0.9051	0.8970	0.8430	0.8515	0.8844	0.8435
√		√			0.8785	0.8769	0.9177	0.8538	0.8532	0.8703	0.8505
√			√		0.8671	0.9172	0.9159	0.8339	0.8414	0.9191	0.8480
√				√	0.8683	0.9204	0.9047	0.8382	0.8568	0.9254	0.8556
	√	√			0.8514	0.8002	0.8833	0.8252	0.8189	0.7914	0.8390
	√		√		0.8483	0.8195	0.8621	0.8001	0.8071	0.8234	0.8422
	√			√	0.8501	0.8536	0.9015	0.8009	0.8334	0.8624	0.8403
		√	√		0.8377	0.8411	0.8552	0.8139	0.8276	0.8397	0.8336
		√		√	0.8478	0.8490	0.8871	0.8269	0.8298	0.8651	0.8514
			√	√	0.7507	0.8002	0.7795	0.6990	0.7390	0.8150	0.7696
√	√	√			0.8793	0.9181	0.9202	0.8659	0.8527	0.8982	0.8513
√	√		√		0.8710	0.9438	0.9046	0.8472	0.8434	0.9360	0.8574
√	√			√	0.8632	0.9399	0.9053	0.8506	0.8669	0.9454	0.8597
√		√	√		0.8701	0.9347	0.9189	0.8481	0.8521	0.9235	0.8599
√		√		√	**0.8912**	0.9250	0.9253	0.8557	**0.8753**	0.9370	0.8727
√			√	√	0.8650	0.9294	0.9095	0.8251	0.8466	0.9330	0.8471
	√	√	√		0.8669	0.8468	0.8962	0.8359	0.8185	0.8520	0.8444
	√	√		√	0.8755	0.8663	0.9127	0.8325	0.8538	0.8820	0.8606
	√		√	√	0.8463	0.8441	0.8794	0.8034	0.8213	0.8546	0.8344
		√	√	√	0.8332	0.8571	0.8964	0.8194	0.8159	0.8630	0.8247
√	√	√	√		0.8903	0.9427	**0.9281**	0.8618	0.8524	0.9394	0.8696
√	√	√		√	0.8900	0.9494	0.9207	0.8670	0.8503	0.9477	**0.8795**
√	√		√	√	0.8646	0.9471	0.9087	0.8431	0.8519	0.9446	0.8577
√		√	√	√	0.8775	0.9354	0.9227	0.8481	0.8482	0.9372	0.8511
	√	√	√	√	0.8728	0.8766	0.9115	0.8289	0.8536	0.8812	0.8536
√	√	√	√	√	0.8909	**0.9513**	0.9267	**0.8764**	0.8574	**0.9502**	**0.8702**

α: miRNA sequence features; β: miRNA disease features; γ: miRNA drug features; δ: miRNA mRNA network features; ε: miRNA mRNA co-localization features.

**Table 6:** Ablation test results on features (performance on seven subcellular localizations measured by AUPR).

Ablation settings					Cytoplasm	Exosome	Nucleolus	Nucleus	Extracellular vesicle	Microvesicle	Mitochondrion
α	β	γ	δ	ε							
√					0.7692	0.9550	0.4510	0.8368	0.4345	0.9424	0.6465
	√				0.7028	0.9150	0.3937	0.7677	0.4308	0.8853	0.6151
		√			0.6997	0.9450	0.3306	0.8002	0.3633	0.9214	0.5929
			√		0.5768	0.9428	0.3527	0.6855	0.3296	0.9236	0.5304
				√	0.5341	0.9519	0.2120	0.6686	0.2800	0.9360	0.5186
√	√				0.7925	0.9789	0.4241	0.8432	0.4739	0.9662	0.6831
√		√			0.8074	0.9699	0.4579	0.8585	0.4636	0.9596	0.7011
√			√		0.7721	0.9786	0.4609	0.8334	0.4376	0.9753	0.6829
√				√	0.7771	0.9815	0.4432	0.8213	**0.4971**	0.9783	0.7013
	√	√			0.7577	0.9524	0.4637	0.8242	0.4499	0.9279	0.6665
	√		√		0.7344	0.9506	0.5321	0.7960	0.3880	0.9397	0.6563
	√			√	0.7378	0.9678	0.4852	0.7894	0.4227	0.9623	0.6786
		√	√		0.7136	0.9541	0.3575	0.8144	0.4189	0.9422	0.6317
		√		√	0.7328	0.9642	0.3915	0.8135	0.4060	0.9577	0.6678
			√	√	0.5816	0.9531	0.3005	0.6864	0.2856	0.9457	0.5107
√	√	√			0.8228	0.9826	0.4543	0.8573	0.4455	0.9713	0.7065
√	√		√		0.8047	0.9880	0.4342	0.8449	0.4747	0.9815	0.6968
√	√			√	0.7891	0.9873	0.5125	0.8532	0.4662	0.9861	0.7183
√		√	√		0.7890	0.9841	**0.5474**	0.8440	0.4776	0.9738	0.7129
√		√		√	0.8176	0.9835	0.4815	0.8537	0.4596	0.9817	0.7384
√			√	√	0.7795	0.9817	0.5046	0.8256	0.4447	0.9758	0.6875
	√	√	√		0.7609	0.9642	0.4992	0.8273	0.4334	0.9528	0.6604
	√	√		√	0.7953	0.9727	0.4651	0.8374	0.4877	0.9663	0.7269
	√		√	√	0.7444	0.9664	0.5053	0.7899	0.4610	0.9565	0.6491
		√	√	√	0.7425	0.9661	0.4360	0.8157	0.4174	0.9549	0.6389
√	√	√	√		**0.8239**	0.9882	0.4545	0.8625	0.4617	0.9828	0.7184
√	√	√		√	0.8220	0.9899	0.4792	0.8605	0.4702	0.9863	**0.7554**
√	√		√	√	0.7709	0.9892	0.4850	0.8392	0.4658	0.9841	0.7148
√		√	√	√	0.7990	0.9839	0.5211	0.8423	0.4601	0.9805	0.7318
	√	√	√	√	0.7778	0.9738	0.5214	0.8194	0.4487	0.9649	0.6904
√	√	√	√	√	0.8192	**0.9905**	0.5298	**0.8763**	**0.4695**	**0.9866**	0.7294

α: miRNA sequence features; β: miRNA disease features; γ: miRNA drug features; δ: miRNA mRNA network features; ε: miRNA mRNA co-localization features.

With above arguments, each feature type was essential for PMiSLocMF. However, their importance may not be same. From the overall performance of classifiers using single feature type (first five rows in Table 4), it was found that the classifier using miRNA sequence features (represented by α in Table 4) generally provided the best performance, followed by that using miRNA disease features (represented by β in Table 4), miRNA drug features (represented by γ in Table 4), miRNA mRNA network features (represented by δ in Table 4), and miRNA mRNA co-localization features (represented by ε in Table 4). This sequence was also the importance sequence for PMiSLocMF to predict miRNA subcellular localizations from the most to the least.

**Figure 5:**
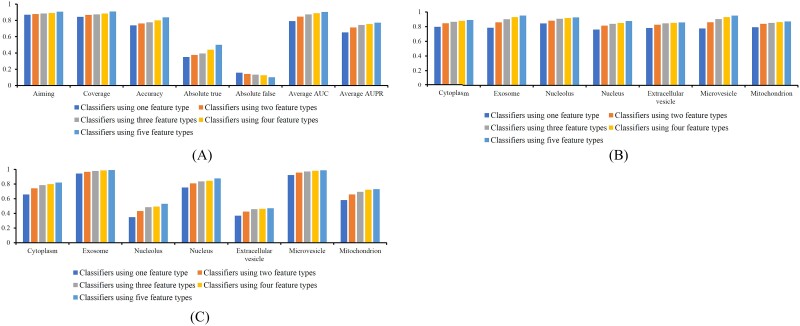
Performance of classifiers using one, two, three, four and five feature types. (A) Overall performance; (B) Performance on seven subcellular localizations measured by AUC; (C) Performance on seven subcellular localizations measured by AUPR. When more feature types are used, the classifiers provide better performance.

It was also interesting to investigate whether more features can improve the performance of classifiers. In view of this, we divided above-mentioned thirty classifiers into four groups: classifiers using one, two, three or four feature types. The PMiSLocMF constituted the fifth group as it used all five feature types. The average aiming, coverage, accuracy, absolute true, absolute false, AUC, and AUPR were counted for each classifier group, which are illustrated in Fig. 5. It was found from Fig. 5(A) that all overall measurements, except absolute false, followed a strictly monotone increasing trend with the addition of feature types, whereas absolute false followed a contrary trend. As for AUC and AUPR values on seven subcellular localizations (Fig. 5(B)-(C)), they also followed a strictly monotone increasing trend with the increment of feature types. All these implied that when more feature types were used, the classifier became better and better.

Finally, we analyzed the relationships between five feature types and seven subcellular localizations. Take the performance of PMiSLocMF on seven subcellular localizations as the baseline (last row in Tables 5 and 6), if the removal of one feature type (i.e., the performance of classifiers with four feature types) induced a sharp drop on the performance on one subcellular localization, this feature type was deemed to be highly related to this subcellular localization. Thus, we looked up the test results in Tables 5 and 6, and set the threshold of decline to 0.02 for easy analysis. Related subcellular localizations for each feature type are listed in Table 7. After taking the common subcellular localizations selected by AUC and AUPR, the following highly related subcellular localizations for some feature types were obtained. For miRNA sequence features, its related subcellular localizations were nucleus and microvesicle. For miRNA disease features, nucleus was the only related subcellular localization. For miRNA drug features, its related subcellular localizations were nucleus and cytoplasm. Such relationships between feature types and subcellular localizations may give insights for improving our model. For example, the further refined miRNA disease features may improve the model’s performance on nucleus.

**Table 7:** Related subcellular localizations of each feature type.

Feature type	Related subcellular localizations selected by AUC	Related subcellular localizations selected by AUPR
miRNA sequence features	Exosome, Microvesicle, Nucleus	Nucleus, Cytoplasm, Mitochondrion, Microvesicle, Extracellular vesicle
miRNA disease features	Nucleus	Nucleus, Cytoplasm
miRNA drug features	Nucleus, Cytoplasm	Cytoplasm, Nucleolus, Nucleus
miRNA mRNA network features	–	Nucleolus
miRNA mRNA co-localization features	–	Nucleolus

### Effectiveness of GATE

The GATE was employed to improve the raw miRNA features derived from miRNA-disease, miRNA-drug, and miRNA–mRNA association networks. It was necessary to investigate whether GATE can really improve the performance of PMiSLocMF. Thus, we removed GATE and rebuilt the classifier. This classifier was also evaluated by 10-fold cross-validation. All measurements of this classifier are listed in Table 8. For easy comparisons, the measurements of PMiSLocMF are also provided in this table. It can be observed that aiming, coverage, accuracy, absolute true, average AUC, and average AUPR all decreased, whereas absolute false increased. In detail, the accuracy and absolute true declined by 0.06 and 0.09, respectively, which were significant decrease. As for the AUC and AUPR values on seven subcellular localizations, they also decreased compared with those of PMiSLocMF. These results suggested that the classifier without GATE was inferior to PMiSLocMF, proving the utility of GATE for building the classifier.

**Table 8:** Ablation test results on graph attention auto-encoder and self-attention layer.

Measurements		Classifier without graph attention auto-encoder	Classifier without self-attention layer	PMiSLocMF
Aiming		0.8821	0.8912	**0.9067**
Coverage		0.8797	0.8870	**0.9087**
Accuracy		0.7736	0.8047	**0.8374**
Absolute true		0.4111	0.4233	**0.5000**
Absolute false		0.1226	0.1176	**0.1012**
Average AUC		0.8942	0.8942	**0.9033**
Average AUPR		0.7545	0.7603	**0.7716**
AUC	Cytoplasm	0.8732	0.8764	**0.8909**
	Exosome	0.9434	0.9468	**0.9513**
	Nucleolus	0.9182	0.9259	**0.9267**
	Nucleus	0.8692	0.8572	**0.8764**
	Extracellular vesicle	0.8482	0.8475	**0.8574**
	Microvesicle	0.9397	0.9497	**0.9502**
	Mitochondrion	0.8678	0.8559	**0.8702**
AUPR	Cytoplasm	0.8052	0.8071	**0.8192**
	Exosome	0.9879	0.9811	**0.9905**
	Nucleolus	0.4683	0.5114	**0.5298**
	Nucleus	0.8679	0.8560	**0.8763**
	Extracellular vesicle	0.4452	0.4603	**0.4695**
	Microvesicle	0.9837	0.9863	**0.9866**
	Mitochondrion	0.7235	0.7197	**0.7294**

### Effectiveness of self-attention layer

The self-attention layer was designed to further improve the quality of features before the final prediction. It was also necessary to investigate its effectiveness in building PMiSLocMF. Thus, we also removed the self-attention layer and thus obtained classifier was assessed by 10-fold cross-validation. The results are listed in Table 8. Compared with the performance of PMiSLocMF, the overall measurements, except absolute false, all decreased. The declines were ~0.01–0.08. The absolute false increased by 0.01. The AUC values on seven subcellular localizations dropped by 0.0005–0.02 and AUPR values decreased by the similar degree. Accordingly, we can conclude that the classifier without self-attention layer was inferior to PMiSLocMF, implying the utility of self-attention layer for improving the performance of PMiSLocMF.

### Deep analysis of the performance of PMiSLocMF on associated diseases, drugs, and mRNAs

**Figure 6:**
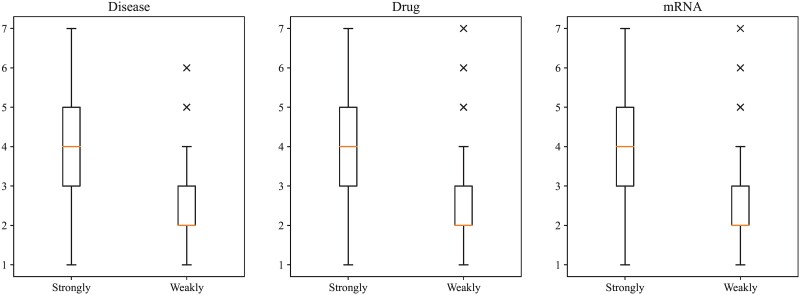
Boxplot to show the label frequencies of miRNAs in different groups. miRNAs in ‘strongly’ group, which are associated with more diseases, drugs, or mRNAs, have more labels (subcellular localizations) and larger label number range.

In this study, we used three association networks (Table 2) to yield essential miRNA features. These networks indicated the associations between miRNAs and three objects (diseases, drugs, and mRNAs). Clearly, the numbers of associated diseases, drugs, and mRNAs for miRNAs were not same. It was interesting to investigate whether the number of associated diseases, drugs, and mRNAs can influence the performance of PMiSLocMF. In view of this, we first ranked the miRNAs with the decreasing order of the numbers of their associated diseases and equally divided all miRNAs into two groups. The first group contained miRNAs that have many associated diseases, whereas the second group consisted of the rest miRNAs that have few associated diseases. The threshold on the number of associated diseases for two miRNA groups was five. These two groups were called ‘strongly’ and ‘weakly’ groups. With the same operations, miRNAs can also be divided into two groups in terms of the numbers of their associated drugs or mRNAs. The thresholds for numbers of associated drugs and mRNAs were two and one, respectively. For the 10-fold cross-validation results of PMiSLocMF, we individually counted five overall measurements (aiming, coverage, accuracy, absolute true, and absolute false) on two groups. These measurements are listed in Table 9. It was amazing that the performance of PMiSLocMF on ‘weakly’ group was evidently better than that of the ‘strongly’ group. In general, the miRNAs in ‘strongly’ group had more associated diseases, drugs, or mRNAs, which provided more informative materials for extracting effective features. Thus, the performance on ‘strongly’ group should be better than that on ‘weakly’ group. However, the test results were on the contrary. In view of this, we further counted the number of labels for miRNAs and drew a box plot to show the label number distribution in each miRNA group, as displayed in Fig. 6. It can be found that miRNAs in ‘strongly’ group generally had more labels, i.e., subcellular localizations, than those in ‘weakly’ group. In detail, the average label numbers for miRNAs in three ‘strongly’ groups were 4.06, 3.96, and 4.04, whereas these numbers for miRNAs in three ‘weakly’ groups were 2.44, 2.55, and 2.46. Furthermore, the span of label numbers for ‘strongly’ miRNA group was also larger than that of ‘weakly’ miRNA group. These facts increased the difficulty in correct prediction of subcellular localizations of miRNAs in ‘strongly’ group. Thus, for the constructed classifier, its prediction on miRNAs with weak associations to other objects was more reliable than those with strong associations. Furthermore, the influences of associated diseases, drugs, and mRNAs on PMiSLocMF were not same. According to Table 9, the gap on absolute true in two groups can be computed. It is easy to obtain that the gap for associated diseases was greatest, followed by that for associated drugs and mRNAs. It was indicated that the number of associated diseases provided the most influence on PMiSLocMF, the number of associated drugs stood at the second place, and the number of associated mRNAs gave the least effects.

**Table 9:** Performance of PMiSLocMF on different miRNA groups.

Associated object	Group	Aiming	Coverage	Accuracy	Absolute true	Absolute false
Disease	Strongly	0.8780	0.8897	0.8051	0.2799	0.1583
	Weakly	**0.9211**	**0.9114**	**0.8444**	**0.6933**	**0.0514**
Drug	Strongly	0.8484	0.9049	0.7715	0.3088	0.1455
	Weakly	**0.9114**	**0.9307**	**0.8455**	**0.5911**	**0.0723**
mRNA	Strongly	0.8919	0.8952	0.8164	0.3613	0.1218
	Weakly	**0.9129**	**0.9159**	**0.8392**	**0.6333**	**0.0884**

### Performance comparison with previous prediction methods

To date, some prediction methods have been proposed to identify miRNA subcellular localizations. Here, we selected the methods built on the same benchmark dataset for a fair comparison, including MiRLoc [41], DAmiRLocGNet [42], and MirLocPredictor [36]. Although the original study on MirLocPredictor adopted a different miRNA dataset, its performance on a similar dataset was reported in [42]. Thus, it was also employed for extensive comparisons. The performance of above three methods is listed in Tables 10 and 11, where the performance of PMiSLocMF is also provided.

**Table 10:** Performance comparison in terms of AUC.

Subcellular localization	MiRLoc [41]	MirLocPredictor^a^ [36]	DAmiRLocGNet [42]	PMiSLocMF
Cytoplasm	0.8366	0.5741	0.8606	**0.8909**
Exosome	0.7395	0.5842	0.7051	**0.9513**
Nucleolus	0.9085	0.5286	**0.9289**	0.9267
Nucleus	0.7765	0.6752	0.7960	**0.8764**
Extracellular vesicle	0.8003	0.6335	0. 8350	**0.8574**
Microvesicle	0.5099	0.5973	0.6757	**0.9502**
Mitochondrion	0.7694	0.6758	0.8332	**0.8702**
Average AUC	0.7630	0.6098	0.8049	**0.9033**

a : The results of this classifier is directly retrieved from [42].

**Table 11:** Performance comparison in terms of AUPR.

Subcellular localization	MiRLoc [41]	MirLocPredictor^a^ [36]	DAmiRLocGNet [42]	PMiSLocMF
Cytoplasm	0.7285	**0.8391**	0.7636	0.8192
Exosome	0.9892	0.8248	0.9248	**0.9905**
Nucleolus	0.4148	0.4925	**0.5739**	0.5298
Nucleus	0.8102	0.4349	0.7961	**0.8763**
Extracellular vesicle	0.2916	0.3434	0.4619	**0.4695**
Microvesicle	0.9203	0.2469	0.8883	**0.9866**
Mitochondrion	0.5277	0.3113	0.6882	**0.7294**
Average AUPR	0.6689	0.4990	0.7281	**0.7716**

a : The results of this classifier is directly retrieved from [42].

The average AUC values of MiRLoc, MirLocPredictor, and DAmiRLocGNet were 0.7630, 0.6098, and 0.8049, respectively (Table 10), which were evidently lower than that of PMiSLocMF (0.9033). Specific to seven subcellular localizations, PMiSLocMF yielded the highest AUC values on six localizations and the AUC on the exceptive localization (nucleolus) stood at the second place. Thus, in terms of AUC, PMiSLocMF was better than MiRLoc, DAmiRLocGNet, and MirLocPredictor. The average AUPR values of MiRLoc, MirLocPredictor, and DAmiRLocGNet were 0.6689, 0.4990, and 0.7281, respectively (Table 11). They were also lower than that of PMiSLocMF, which was 0.7716. For AUPR values on seven subcellular localizations, PMiSLocMF occupied the first places on five localizations and the second places on two localizations. Generally, PMiSLocMF was also superior to MiRLoc, DAmiRLocGNet, and MirLocPredictor in terms of AUPR. The superiority was not very evident compared with that by considering AUC. Anyway, it can be concluded that PMiSLocMF was better than MiRLoc, DAmiRLocGNet, and MirLocPredictor for the prediction of miRNA subcellular localizations.

The special advantages of PMiSLocMF existed, which induced better performance than other three previous methods. The PMiSLocMF adopted more complete properties of miRNAs than MiRLoc, DAmiRLocGNet, and MirLocPredictor. MirLocPredictor only used the miRNA sequence information, MiRLoc adopted the miRNA–mRNA association information and mRNA subcellular localizations, DAmiRLocGNet used the miRNA sequence and miRNA-disease association information. PMiSLocMF employed all above information and further fused the miRNA-drug association information, which was first used to predict miRNA subcellular localizations. Furthermore, the powerful feature construction methods (node2vec and GATE) were used for extracting essential features of miRNAs, which gave a great help for making correct predictions.

### Performance of PMiSLocMF on the independent test dataset

**Figure 7:**
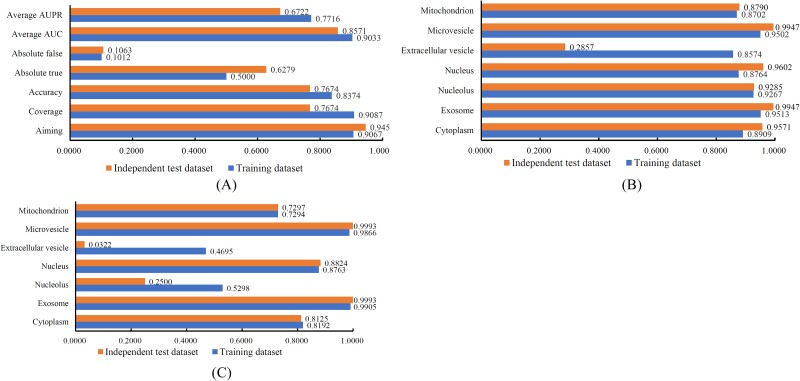
Bar chart to compare the performance of PMiSLocMF on training and independent test datasets. (A) Bar chart on seven overall measurements; (B) Bar chart on AUC values on seven subcellular localizations; (C) Bar chart on AUPR values on seven subcellular localizations. The performance of PMiSLocMF on training and independent test datasets is quite similar on most measurements.

An independent test dataset containing 41 miRNAs was constructed as mentioned in Section Benchmark dataset. The model PMiSLocMF trained on dataset *S* was applied to this independent dataset to examine the generalization ability of PMiSLocMF. Seven overall measurements, AUC and AUPR values on seven subcellular localizations are shown in Fig. 7. For easy comparison, same measurements of PMiSLocMF on training dataset *S* are also illustrated in this figure. It can be found that PMiSLocMF provided similar performance on most overall measurements (Fig. 7(A)). As for AUC and AUPR values on seven subcellular localizations, PMiSLocMF yielded similar values on all localizations except the AUC on one localization (extracellular vesicle) (Fig. 7(B)) and AUPR on two localizations (extracellular vesicle and nucleolus) (Fig. 7(C)). These results suggested that PMiSLocMF had similar performance on the training and independent test datasets, proving the strong generalization ability of PMiSLocMF.

### Limitations of this study

Although the proposed model PMiSLocMF provided high performance in predicting miRNA subcellular localizations, some limitations also existed. First, this model was tested only on human miRNAs. Its performance on other species or across species was not examined. Second, we employed several miRNA properties for accessing more complete miRNA representations. However, such operation led to an application limitation of our model. For some miRNAs, not all properties were available. In this case, the model cannot perfectly predict the subcellular localizations of these miRNAs. Third, the investigated human miRNA dataset *S* was evidently imbalanced. We did not employ over-sampling or under-sampling methods to tackle this problem, inducing imbalanced performance on some localizations. In future, we will continue this work to overcome above limitations for designing more efficient models with wide applications.

## Conclusion

This study proposed a novel multi-label classifier for predicting miRNA subcellular localizations. This classifier fused several miRNA properties and employed or designed feature extraction schemes to generate essential miRNA features from these properties. The cross-validation results indicated the good performance of the classifier and it was superior to all existing methods based on the same dataset. The ablation tests shown that more miRNA properties were helpful to improve the classifier, and GATE and self-attention layer gave key contributions. We also found that the classifier can give correct predictions for miRNAs with weak associations to diseases, drugs, or mRNAs. It is hopeful that the proposed classifier can be a useful tool in determining the subcellular localizations of miRNAs. The dataset and codes are available at https://github.com/Gu20201017/PMiSLocMF.

**Key Points**

- A novel computational method is designed to identify miRNA subcellular localizations.
- This method adopts a full representation of the miRNA, which is derived from several miRNA properties.
- The method provides high performance and is superior to previous methods.
- More properties of miRNAs can lead to better performance and their associations with diseases, drugs, and mRNAs influence the performance of the method.

## Supplementary Material

Supp_I_bbae386

Table_S1_bbae386

## Data Availability

The miRNA dataset is available at RNALocate database (http://www.rnalocate.org/). All data and codes are available at https://github.com/Gu20201017/PMiSLocMF.
