# Supplementary material for: PMiSLocMF: predicting miRNA subcellular localizations by incorporating multi-source features of miRNAs
Source: Brief Bioinform. 2024 Aug 18;25(5):bbae386. doi: 10.1093/bib/bbae386 (PMC11330342; doi:10.1093/bib/bbae386)
Supplement: Supp_I_bbae386 [file supp_i_bbae386.docx]

**Supplementary Material I.** Details of methods, evaluation metrics and parameter setting.

## Node2vec

Node2vec is a powerful algorithm that can embed nodes in a network into a low-dimensional vector space while preserving structural information between nodes. The core idea of node2Vec involves random walks between neighboring nodes, generating substantial node sequences through multiple iterations of random walks. These sequences capture both the proximity relationships and semantic connections among nodes in the given network. Unlike Deepwalk [1], another network embedding algorithm which employs a fully random walk to generate node sequences, node2vec designs a specific sampling strategy. For each node in the network, node2vec generates *m* paths with length *l* starting from this node using a well-designed sampling strategy. Suppose there is a path starting at node $c_{0}=y$ and it has been extended to the *i*-th node $c_{i-1}=v$. The following equation defines the possibility of the next node $c_{i}=x$

| $P\left( c_{i}=x\mid c_{i-1}=v \right)=\left\{ \begin{aligned} & \frac{\pi_{vx}}{Z} &&\text{ if }\text{v}\text{ and }x are adjacent \\ & 0 &&\text{ otherwise } \end{aligned} \right.$, | (1) |
| --- | --- |

where $\pi_{vx}$ is the unnormalized transition probability between *v* and *x*, and *Z* is the normalization constant, which is defined the sum of transition probabilities between *v* and other nodes. $\pi_{vx}$ is a pivotal factor influencing the quality of generated paths. It is determined by two parameters *p* and *q*. Parameter *p*, called return parameter, controls the likelihood of returning to the previous node, influencing depth-first exploration; whereas parameter *q*, also called in-out parameter, controls the probability of moving to neighboring nodes, influencing breadth-first exploration. Suppose that the (*i*-1)-th node of the current path is *t*, i.e., $c_{i-2}=t$. $\pi_{vx}$ is defined as follows:

| $\pi_{vx}=\alpha_{pq}(t,x)\cdot w_{vx}$, | (2) |
| --- | --- |

where $w_{vx}$ is the weight of edge $(v,x)$ and $\alpha_{pq}(t,x)$ is computed by

| $\alpha_{pq}(t,x)=\left\{ \begin{aligned} &\frac{1}{p} &&\text{ if }d_{tx}=0 \\ &1 &&\text{ if }d_{tx}=1 \\ &\frac{1}{q} &&\text{ if }d_{tx}=2 \end{aligned} \right.$, | (3) |
| --- | --- |

where $d_{tx}$ represents the distance from *t* to *x*. The current path stops to extend until its length reaches *l*. After the paths starting from each node have been generated, the node sequences in obtained paths are deemed as sentences and nodes are regarded as words. The word2vec with SkipGram is applied to this information for generating the feature vector of each node.

## Graph attention auto-encoder

Graph attention auto-encoder (GATE) contains encoder and decoder procedures. The encoder updates the raw representation of each node by considering the representations of its neighbors. The decoder tries to recover the raw representations of nodes as perfect as possible.

Generally, there are several layers in the encoder procedure. The raw representation of the *i*-th node is denoted by $x_{i}=h_{i}^{(0)}$ and its new representation after the *k*-th layer is represented by $h_{i}^{(k)}$, which is obtained by a well-designed aggregation scheme from its neighbors. This scheme considers the importances of its neighbors, which is solved by a self-attention mechanism. In the *k*-th layer, the relevance between the *i*-th node and its neighbor, say the *j*-th node, is expressed as follows:

| $e_{ij}^{(k)}=\mathrm{Sigmoid}\left( v_{s}^{(k)^{T}}\sigma\left( W^{(k)}h_{i}^{(k-1)} \right)+v_{r}^{(k)^{T}}\sigma\left( W^{(k)}h_{j}^{(k-1)} \right) \right)$, | (4) |
| --- | --- |

where $W^{(k)}\in\mathbb{R}^{d^{(k)}\times d^{(k-1)}}$, $v_{s}^{(k)}\in\mathbb{R}^{d^{(k)}}$, and $v_{r}^{(k)}\in\mathbb{R}^{d^{(k)}}$ are the trainable parameters. $\sigma$ is the activation function, sigmoid stands for the sigmoid function. The softmax function follows to normalize the outcomes of **Eq. 4**, formulated by

| $\alpha_{ij}^{(k)}=\frac{\exp\left( e_{ij}^{(k)} \right)}{\sum_{l\in\mathcal{N}_{i}} \exp\left( e_{il}^{(k)} \right)}$, | (5) |
| --- | --- |

where $\alpha_{ij}^{(k)}$ represents the attention coefficient of the *j*-th node relative to *i*-th node in the *k*-th encoder layer, and $\mathcal{N}_{i}$ denotes the closed neighborhood of the *i*-th node. Then, the new representation of the *i*-th node in the *k*-th encoder layer is computed by

| $h_{i}^{(k)}=\sum_{j\in\mathcal{N}_{i}} \alpha_{ij}^{(k)}\sigma\left( W^{(k)}h_{j}^{(k-1)} \right)$, | (6) |
| --- | --- |

The output of the last encoder layer is picked up as the final new representations of nodes. For the *i*-th node, this representation is denoted by $h_{i}$. If there are *L* layers in the encoder procedure, $h_{i}=h_{i}^{(L)}$.

The decoder procedure contains same number of layers in the encoder procedures. Its purpose is to recover $x_{i}$ from $h_{i}$. Thus, the output of encoder procedure, $h_{i}$, is the input of the decoder procedure, which was denoted by $\hat{h}_{i}^{(L)}$, i.e., $\hat{h}_{i}^{(L)}=h_{i}$. In the *k*-th decoder layer, the attention coefficient of the *j*-th node relative to *i*-th node is computed by

| $\hat{e}_{ij}^{(k)}=\mathrm{Sigmoid}\left( \hat{v}_{s}^{(k)^{T}}\sigma\left( \hat{W}^{(k)}\hat{h}_{i}^{(k)} \right)+\hat{v}_{r}^{(k)^{T}}\sigma\left( \hat{W}^{(k)}\hat{h}_{j}^{(k)} \right) \right)$, | (7) |
| --- | --- |
| $\hat{\alpha}_{ij}^{(k)}=\frac{\exp\left( \hat{e}_{ij}^{(k)} \right)}{\sum_{l\in\mathcal{N}_{i}} \exp\left( \hat{e}_{il}^{(k)} \right)}$ | (8) |

where $\hat{W}^{(k)}\in\mathbb{R}^{d^{(k-1)}\times d^{(k)}}$, $\hat{v}_{s}^{(k)}\in\mathbb{R}^{d^{(k-1)}}$, and $\hat{v}_{r}^{(k)}\in\mathbb{R}^{d^{(k-1)}}$ are the trainable parameters in the *k*-th decoder layer, $\sigma$ and sigmoid are same as those in **Eq. 4**, and $\mathcal{N}_{i}$ is same as that in **Eq. 5**. Then, the representation of the *i*-th node after the *k*-th decoder layer is updated by

| $\hat{h}_{i}^{(k-1)}=\sum_{j\in\mathcal{N}_{i}} \hat{\alpha}_{ij}^{(k)}\sigma\left( \hat{W}^{(k)}\hat{h}_{j}^{(k)} \right)$ | (9) |
| --- | --- |

The output of the last decoder layer is pick up as the ultimately reconstructed node representation. For the *i*-th node, this representation is denoted by $\hat{x}_{i}$, i.e., $\hat{x}_{i}=\hat{h}_{i}^{(0)}$.

To assess the quality of the output of encoder procedure, i.e., $h_{i}$ for the *i*-th node, GATE employs the loss function, which contains the following two parts:

| $\sum_{i=1}^{N} \left\Vert x_{i}-\hat{x}_{i} \right\Vert_{2}$, | (10) |
| --- | --- |
| $-\sum_{i=1}^{N} \sum_{j\in\mathcal{N}_{i}} \log\left( \frac{1}{1+\exp\left( -h_{i}^{T}h_{j} \right)} \right)$, | (11) |

where *N* is the number of nodes. **Eq. 10** represents the loss of the raw and reconstructed representations. **Eq. 11** indicates the similarity of the representations of one node and its neighbors. Generally, adjacent nodes should be assigned similar representations. By combining **Eqs. 10** and **11**, the final loss function is as follows:

| $\text{Loss }=\sum_{i=1}^{N} \left\Vert x_{i}-\hat{x}_{i} \right\Vert_{2}-\lambda\sum_{j\in\mathcal{N}_{i}} \log\left( \frac{1}{1+\exp\left( -h_{i}^{T}h_{j} \right)} \right)$, | (12) |
| --- | --- |

where $\lambda$ is a parameter used to control the weight of two loss parts.

## Self-attention layer

In the self-attention layer, self-attention mechanisms learn the weights between features for better representing the internal structure of the input features, which can help the model capture complex dependencies between features. Three weight matrices $W_{Q}$, $W_{K}$, and $W_{V}$ are employed in this procedure for linear transformations on the input features, yielding $Q$, $K$, and $V$ as follows:

| $\left\{ \begin{aligned} Q=X\cdot W_{Q} \\ K=X\cdot W_{K} \\ V=X\cdot W_{V} \end{aligned} \right.$, | (13) |
| --- | --- |

where $X$ represents the input feature vector. Then, the attention score matrix $A$ is computed by

| $A=\frac{QK^{T}}{\sqrt{d_{k}}}$, | (14) |
| --- | --- |

where $d_{k}$ stands for the dimension of $Q$ or $K$. A softmax operation is applied to $A$ to produce the attention weight matrix $M$, i.e, $M=softmax(A)$. Finally, the output $Y$ of the self-attention layer is obtained by $V$ and $M$ using the following equation

| $Y=MV$ | (15) |
| --- | --- |

## Evaluation metrics

Cross-validation is a commonly used method to assess the performance of classifiers [2]. In this method, samples are divided into several parts. Each part is picked up as test set one by one, whereas the rest parts comprise the training set. The classifier based on the training set is applied to the test set. Thus, each sample is tested exactly once. Generally, samples are divided into five or ten parts. Here, we adopted 10-fold cross-validation, i.e., samples were divided into ten parts, to evaluate the performance of classifiers.

For multi-label classification problems, several overall measurements have been designed to evaluate the quality of predicted results. Here, we employed five measurements, including aiming, coverage, accuracy, absolute true, and absolute false [3]. These measurements have wide applications in measuring the performance of multi-label classifiers [4-13]. Some notations are necessary to clearly introduce these measurements. Let $\mathbb{L}_{k}$ represent the subset containing the observed labels of the *k*-th sample and $\mathbb{L}_{k}^{*}$ denote the subset containing the predicted labels of the *k*-th sample. Then, above five measurements are computed by

| $\left\{ \begin{aligned} &\text{ }\text{Aiming}=\frac{1}{N}\sum_{k=1}^{N} \left( \frac{\left\Vert\mathbb{L}_{k}\cap\mathbb{L}_{k}^{*} \right\Vert}{\left\Vert\mathbb{L}_{k}^{*} \right\Vert} \right) \\ &\text{ Coverage}=\frac{1}{N}\sum_{k=1}^{N} \left( \frac{\left\Vert\mathbb{L}_{k}\cap\mathbb{L}_{k}^{*} \right\Vert}{\left\Vert\mathbb{L}_{k} \right\Vert} \right) \\ &\text{ }\text{Accuracy}=\frac{1}{N}\sum_{k=1}^{N} \left( \frac{\left\Vert\mathbb{L}_{k}\cap\mathbb{L}_{k}^{*} \right\Vert}{\left\Vert\mathbb{L}_{k}\cup\mathbb{L}_{k}^{*} \right\Vert} \right) \\ &\text{ }\text{Absolute true}=\frac{1}{N}\sum_{k=1}^{N} \Delta\left( \mathbb{L}_{k},\mathbb{L}_{k}^{*} \right) \\ &\text{ }\text{Absolute false}=\frac{1}{N}\sum_{k=1}^{N} \left( \frac{\left\Vert\mathbb{L}_{k}\cup\mathbb{L}_{k}^{*} \right\Vert-\left\Vert\mathbb{L}_{k}\cap\mathbb{L}_{k}^{*} \right\Vert}{M} \right) \end{aligned} \right.$, | (16) |
| --- | --- |

where $N$ is the total number of samples (miRNAs in this study), $M$ is the total number of labels (*M* = 7 in this study), $\Delta\left( \mathbb{L}_{k},\mathbb{L}_{k}^{*} \right)$ is defined as

| $\Delta\left( \mathbb{L}_{k},\mathbb{L}_{k}^{*} \right)=\left\{ \begin{aligned} &1, &&\text{ if all labels in }\mathbb{L}_{k}\text{ are identical to those in }\mathbb{L}_{k}^{*} \\ &0, &&\text{ otherwise } \end{aligned} \right.$ | (17) |
| --- | --- |

According to **Eq. 16**, aiming is the average proportion of correctly predicted labels among all predicted labels, which is similar to precision in binary classification. Coverage is the average proportion of correctly predicted labels among all observed labels, similar to recall in binary classification. Accuracy is similar to the accuracy on single-label classification, indicating the average proportion of correctly predicted labels among all predicted and observed labels. Absolute true is a more strict measurement, standing for the proportion of the completely correctly predicted samples. As for absolute false, also called Hamming loss, it indicates the average of proportion of incorrectly predicted labels among all labels. All above measurements are between 0 and 1. The higher the aiming, coverage, accuracy, and absolute true, the higher the performance of the classifier; whereas absolute false is on the contrary.

Besides the overall measurements, we also used individual measurements on each label to partly evaluate the performance of classifiers. For one label, samples with this label are termed as positive samples, whereas others are regarded as negative samples. Then, the popular receiver operating characteristic (ROC) and precision-recall (PR) curves can be obtained for each label. The area under these two curves are important measurements, denoted as AUC and AUPR in this study. These two measurements were also used in previous studies [14, 15], which were used here for easy comparisons. The average AUC and AUPR on all labels were also counted in this study to give an overall evaluation of the classifier.

## Parameter setting

To build a classifier with the performance as high as possible, some key parameters should be tuned. It is known that the representations of miRNAs are essential for the correct prediction of subcellular localizations. According to **Figure 3**, node2vec and GATE played important roles in generating miRNA features. The parameters of these two methods were tuned as follows.

There are several parameters in node2vec. We tuned the following parameters for improving the performance of PMiSLocMF.

(I) Embedding dimension (*d*). We tried three values for this parameter: 64, 128, and 256. For the miRNA sequence similarity network, 64-D feature vectors yield the best performance of PMiSLocMF. As for the miRNA-disease, miRNA-drug, miRNA-mRNA association networks, the optimal performance was obtained when this parameter was set to 128.

(II) Random walk length (*l*). To better explore deep structures in the networks and capture global patterns and inter-node relationships, we set this parameter to a large value as 150.

(III) Number of random walks (*m*). To generate enough node sequences, thereby providing more information for embedding learning, this parameter was also set to a high value as 200.

(IV) Return parameter (*p*) and in-out parameter (*q*). These two parameters were set to their default values (*p*=*q*=1).

Some key parameters are contained in GATE, which were tuned as below.

(I) Number of layers. Based on the original research on GATE [16] and some previous studies [17, 18], we set this parameter to 2, i.e., 2 encoder and 2 decoder layers were contained in GATE.

(II) Number of neurons in layers. The number of neurons in decoder layers should match the corresponding encoder layers. We initially set 128 and 64 neurons in two encoder layers and progressively increased them. The classifier provided the best performance when the encoder layers contained 256 and 128 neurons.

(III) Learning rate. Some values of this parameter were attempted, including 10^-2^, 10^-3^, and 10^-4^. When the learning rate was set to 10^-3^ and 10^-4^, the GATE converged too slowly and the classifier’s performance was not satisfied. Therefore, the learning rate of GATE was set to 10^-2^.

(IV) Parameter $\lambda$ in loss function. It was set to its default value (1).

(V) Binarization threshold. When constructing the miRNA functional similarity network $N_{F}$, we set a binarization parameter (*T*) to discard weak linkages between miRNAs and retain strong linkages. This parameter was set to various values between 0.5 and 0.9. As the miRNA functional similarity network was both used for improving raw miRNA features derived from miRNA-disease, miRNA-drug, and miRNA-mRNA association networks and the binarization threshold may be different for different features, we used grid search to yield the optimal binarization thresholds for features derived from different association networks. As a result, the AUC and AUPR all reached the highest when the threshold was set to 0.8. Thus, this parameter was set to 0.8.

In addition, the prediction procedure used self-attention and fully connected layers. In the self-attention layer, the sizes of three matrices $W_{Q}$, $W_{K}$, and $W_{V}$ were all set to 452🞨452. Two hidden layers were contained in the fully connected layer. The first hidden layer contained 64 neurons, whereas the second layer included 32 neurons.

The detail parameter setting of PMiSLocMF is listed in **Table S1**.

**References**

1. Perozzi B, Al-Rfou R, Skiena S. Deepwalk: Online learning of social representations. In: Proceedings of the 20th ACM SIGKDD international conference on Knowledge discovery and data mining. 2014, p. 701-710.

2. Kohavi R. A study of cross-validation and bootstrap for accuracy estimation and model selection. In: International joint Conference on artificial intelligence. 1995, p. 1137-1145. Lawrence Erlbaum Associates Ltd.

3. Chou KC. Some remarks on predicting multi-label attributes in molecular biosystems, Mol Biosyst 2013;9:1092-1100.

4. Chen L, Li L. Prediction of Drug Pathway-based Disease Classes using Multiple Properties of Drugs, Current Bioinformatics 2024.

5. Yan C, Suo Z, Wang J et al. DACPGTN: Drug ATC Code Prediction Method Based on Graph Transformer Network for Drug Discovery, Front Pharmacol 2022;13:907676.

6. Zhou J-P, Chen L, Guo Z-H. iATC-NRAKEL: An efficient multi-label classifier for recognizing anatomical therapeutic chemical classes of drugs, Bioinformatics 2020;36:1391-1396.

7. Zhou J-P, Chen L, Wang T et al. iATC-FRAKEL: A simple multi-label web-server for recognizing anatomical therapeutic chemical classes of drugs with their fingerprints only, Bioinformatics 2020;36:3568-3569.

8. Wang X, Wang Y, Xu Z et al. ATC-NLSP: Prediction of the Classes of Anatomical Therapeutic Chemicals Using a Network-Based Label Space Partition Method, Front Pharmacol 2019;10:971.

9. Cheng X, Zhao SG, Xiao X et al. iATC-mHyb: a hybrid multi-label classifier for predicting the classification of anatomical therapeutic chemicals, Oncotarget 2017;8:58494-58503.

10. Cheng X, Zhao S-G, Xiao X et al. iATC-mISF: a multi-label classifier for predicting the classes of anatomical therapeutic chemicals, Bioinformatics 2016;33:341-346.

11. Chen L, Chen Y. RMTLysPTM: Recognizing multiple types of lysine PTM sites by deep analysis on sequences, Briefings in Bioinformatics 2024;25:bbad450.

12. Chen L, Qu R, Liu X. Improved multi-label classifiers for predicting protein subcellular localization, Mathematical biosciences and Engineering 2024;21:214-236.

13. Chen L, Zhang C, Xu J. PredictEFC: a fast and efficient multi-label classifier for predicting enzyme family classes, BMC Bioinformatics 2024;25:50.

14. Xu M, Chen Y, Xu Z et al. MiRLoc: predicting miRNA subcellular localization by incorporating miRNA-mRNA interactions and mRNA subcellular localization, Brief Bioinform 2022;23.

15. Bai T, Yan K, Liu B. DAmiRLocGNet: miRNA subcellular localization prediction by combining miRNA-disease associations and graph convolutional networks, Brief Bioinform 2023;24.

16. Salehi A, Davulcu H. Graph attention auto-encoders, arXiv preprint 2019.

17. Deng L, Liu Z, Qian Y et al. Predicting circRNA-drug sensitivity associations via graph attention auto-encoder, BMC Bioinformatics 2022;23:160.

18. Yang B, Chen H. Predicting circRNA-drug sensitivity associations by learning multimodal networks using graph auto-encoders and attention mechanism, Brief Bioinform 2023;24:bbac596.
