# Supplementary material for: PMiSLocMF: predicting miRNA subcellular localizations by incorporating multi-source features of miRNAs
Source: Brief Bioinform. 2024 Aug 18;25(5):bbae386. doi: 10.1093/bib/bbae386 (PMC11330342; doi:10.1093/bib/bbae386)
Supplement: Table_S1_bbae386 [file table_s1_bbae386.docx]

**Table S1.** Parameter setting of PMiSLocMF.

| **Method** | **Parameter** | **Setting** |
| --- | --- | --- |
| Node2vec | Embedding dimension (*d*) | 64 (miRNA sequence similarity network)  128 (miRNA-disease, miRNA-drug, miRNA-mRNA association networks) |
|  | Random walk length (*l*) | 150 |
|  | Number of random walks (*m*) | 200 |
|  | Return parameter (*p*) | 1 |
|  | In-out parameter (*q*) | 1 |
| Graph attention auto-encoder | Layers in encoder and decoder | 2 |
|  | Number of neurons in layers | 256 (first encoder layer)  128 (second encoder layer) |
|  | Learning rate | 10^-2^ |
|  | $\lambda$ | 1 |
|  | Binarization threshold (*T*) | 0.8 |
| Self-attention layer | Sizes of trainable weight matrices ($W_{Q}$, $W_{K}$, and $W_{V}$) | 452🞨452 |
| Fully connected layer | Number of hidden layers | 2 |
|  | Number of neurons in hidden layers | 64 (first hidden layer)  32 (second hidden layer) |
